# Suicide completion in secondary mental healthcare: a comparison study between schizophrenia spectrum disorders and all other diagnoses

**DOI:** 10.1186/s12888-014-0213-z

**Published:** 2014-08-01

**Authors:** Javier-David Lopez-Morinigo, Andrea C Fernandes, Chin-Kuo Chang, Richard D Hayes, Matthew Broadbent, Robert Stewart, Anthony S David, Rina Dutta

**Affiliations:** Department of Psychosis Studies, King’s College London, Institute of Psychiatry, De Crespigny Park, PO Box 68, London, SE5 8AF UK; Department of Psychological Medicine, King’s College London, Institute of Psychiatry, London, UK

**Keywords:** Suicide completion, Secondary mental healthcare, Schizophrenia spectrum disorders

## Abstract

**Background:**

Suicide completion is a tragic outcome in secondary mental healthcare. However, the extent to which demographic and clinical characteristics, suicide method and service use-related factors vary across psychiatric diagnoses remains poorly understood, particularly regarding differences between ‘schizophrenia spectrum disorders (SSD)’ and ‘all other diagnoses’, which may have implications for suicide prevention in high risk groups.

**Methods:**

308 patients who died by suicide over 2007–2011 were identified from the South London and Maudsley NHS Foundation Trust Biomedical Research Centre Case Register. Demographic, clinical, services use-related factors, ‘full risk assessment’ ratings and the Health of the Nation Outcome Scale (HONOS) scores were compared across psychiatric diagnoses. Specifically, differences between patients with and without SSD were investigated.

**Results:**

Patients with SSD ended their lives at a younger age, were more frequently of Black ethnicity and had higher levels of social deprivation than other diagnoses. Also, these patients were more likely to have HONOS and ‘risk assessment’ completed. However, patients who had no SSD scored significantly higher on ‘self-injury’ and ‘depression’ HONOS items and they were more likely to have the following ‘risk assessment’ items: ‘suicidal ideation’, ‘hopelessness’, ‘feeling no control of life’, ‘impulsivity’ and ‘significant loss’. Of note, ‘disengagement’ was more common in patients with SSD, although they had been seen by the staff closer to the time of suicide than in all-other diagnoses. Whilst ‘hanging’ was the most common suicide method amongst patients with non-SSD, most service users with a SSD diagnosis used ‘jumping’ (from heights or in front of a vehicle).

**Conclusions:**

Suicide completion characteristics varied between SSD and other diagnoses in patients receiving secondary mental healthcare. In particular, although clinicians tend to more frequently recognize suicide risk as a focus of concern in patients who have SSD, who are therefore more likely to have a detailed risk assessment documented; ‘known’ suicide risk factors appear to be more relevant in patients with non-SSD. Hence, the classic suicide prevention model might be of little use for SSD.

## Background

Suicide is a serious international public health problem. Every year almost one million people die by suicide around the world [1]. Of note, suicide has become especially concerning among young people as one of the three leading causes of death in the most economically productive age group (15–44 years) and the second leading cause of death in 15–19 year olds [2]. Since 2008, suicide prevention has become a major priority within World Health Organization mental health policies [3]. However, suicide prevention strategies have yielded mixed results so far (see [4]).

Recent literature on suicide seems to support a ‘suicidal spectrum’ ranging from suicidal ideation to suicide completion, with decreasing prevalence and increasing lethality [5]. With regard to suicide completion, psychological autopsy studies have revealed that about 90% of people who killed themselves had a ‘psychiatric disorder’, contributing to 47-74% of the population risk of suicide, and with half of suicide completers meeting criteria for depression [6,7]. In other words, the presence of a psychiatric condition appears to be the strongest risk known factor for suicide [6]. It could therefore be envisaged that better management of mental disorders might reduce suicide rates. More specifically, secondary mental health services may play a crucial role in ‘suicide prevention’ [1].

While a list of risk factors have been strongly associated with ‘suicidal behaviour’, it remains unclear the extent to which such ‘known’ risk factors vary across psychiatric diagnoses [8]. In particular, a better understanding of associations between risk factors and diagnoses may lead to the development and implementation of diagnosis-driven suicide prevention strategies in secondary mental health services. For instance, limiting access to instruments of suicide has been demonstrated to reduce suicide rates at a population level (e.g. [9,10]). However, it remains unclear whether there are specific associations of psychiatric diagnoses with suicide completion methods [11].

Suicide has been found to be the largest single cause of excess mortality in schizophrenia [12,13]. However, the rate of suicide in schizophrenia has been recently demonstrated to be lower, from 2% [14] to 5% [15], than the ‘classic’ figure of 10% [16,17]. Of note, while some recognised general suicide risk factors have been replicated in schizophrenia patients such as being male, living alone and hopelessness; specific suicide risk factors have also been linked to schizophrenia such as lower treatment adherence [18]. Patients with schizophrenia tend not to report suicidal ideation [19], which is a common suicide risk factor for most other psychiatric conditions [4]. Also, suicide risk assessment in schizophrenia remains under-utilized [20]. It therefore seems that the classic suicide prevention model has been less helpful in schizophrenia spectrum disorders than in other diagnoses. Specific suicide completion characteristics in patients with schizophrenia and related disorders in comparison to other diagnoses have not been investigated as fully.

In the UK, over the last two decades the Department of Health has aimed to reduce suicides at a national level [21]. In keeping with this, instruments such as the Health of the Nation Outcome Scale (HoNOS) [22] and structured clinical risk assessments have been strongly recommended [23] and widely used by mental health teams. The extent to which the ‘known’ risk factors for suicide evaluated by such instruments vary across psychiatric diagnoses in patients who took their lives, is also unknown.

We aimed to investigate differences across diagnoses in a sample of patients receiving secondary mental healthcare from teams supervised by Consultant Psychiatrists (i.e. not those treated solely by general practitioners (family physicians) in primary care) who went on to die from suicide. Specifically, sociodemographic and clinical variables, including HoNOS and ‘risk assessment’ ratings and ‘service use’-related factors, and suicide methods were compared between patients with and without schizophrenia spectrum disorders (SSD) who all died by suicide. Hence, our methodology was not designed to test potential suicide risk factor differences across diagnoses since all the participants had the same fatal outcome. Instead our research question was to investigate differences between patients with and without SSD in a sample of secondary mental health service users who all ended their lives.

## Methods

### Sample

The sample comes from the South London and Maudsley (SLaM) Biomedical Research Centre (BRC) Case Register. SLaM is an NHS Trust which provides secondary mental health care to four boroughs in South-East London (UK): Lambeth, Southwark, Lewisham and Croydon. Approximately 1.23 million inhabitants reside in this geographic catchment area, which as a whole was found to be comparable with other populations of London in terms of age, gender, education and socio-economic status distributions [24]. Fully-electronic health records have been in use across all SLaM services since 2006, and in 2007–08 the Clinical Record Interactive Search (CRIS) system was built which renders de-identified copies of these available for research use with appropriate governance structures [24]. Under UK law, anonymised data can be analysed without prior consent, and CRIS has received ethical approval in this respect as a data resource for secondary analyses from the Oxfordshire Research Ethics Committee C (reference: 08/H0606/71 + 5), currently accessing data on over 250,000 patients. The same Research Ethics approval also covers the pseudonymised linkage between CRIS data and those from the Office for National Statistics (ONS) in March 2012 [25] which registers all deaths in the UK and the official cause of death, including suicide and the method of suicide according to ICD-10 classification [26].

The study sample for our analysis was composed of those patients who were ‘active’ to SLaM (i.e. had at least one face-to-face contact with a clinical member of staff) at any point before 31st December 2011, and who had died by suicide (according to the ONS [25] certificate of death) during the period 1st January 2007 to 31st December 2011, including those with an ‘undetermined cause of death’ (ICD-10 Y codes) [26]. Of note, in the UK most ‘open verdicts’ are very likely to be deaths by suicide as the coroner, who under UK law determines the cause of death, is required to provide evidence of suicide intent ‘beyond reasonable doubt’ [27].

### Measures

#### Demographic and clinical variables

Date of birth, gender, ethnicity, ‘hearing, visual or mobility impairments’ (recorded as present/absent) and ICD-10 diagnosis are compulsory fields in the source clinical records system and were analysed. Social deprivation was estimated with the ‘Index of Multiple Deprivation’ (IMD). In particular, area-level deprivation scores were available through an anonymous link created in CRIS between lower super output area residence code of the latest permanent address (a geographic unit comprising approximately 400 households) and summary data for that area from 2001 UK Census output. The Index of Multiple Deprivation is derived from seven domains: income, employment, health, education, housing and services, crime and environment [28].

#### Diagnoses

Distributions and labelling of ICD-10 [26] primary diagnoses in the sample are summarized in Table 1. In particular, ICD-10 diagnoses [26] were made by the treating consultant psychiatrist, including the use of the OPCRIT system [29] for those patients with psychotic disorders, which has been demonstrated to have high reliability across the Trust psychiatrists [30], who are mandatorily trained in the use of this tool. As a result, those patients who were just seen on one occasion such as a single presentation to A&E or one outpatient appointment were more likely not to be diagnosed as reported below. For analyses described here, categories were hierarchically created from ICD-10 codes [26]. Thus, only one of the following exclusive diagnostic categories were recorded if the patient had received more than one ICD-10 primary diagnosis: ‘F2’ – ‘schizophrenia spectrum disorder’ (SSD); ‘F31 and F32.3’ – ‘Affective Psychosis’; ‘F32.1, F32.2, F33, F34 and F4’ – ‘Mood disorder/Neuroses’; ‘F0 and F1’ – ‘Organic/Drugs’; ‘F5, F6 and the remaining categories’ were grouped as ‘All other diagnoses’. 94 remaining patients (30.5%) were labelled as ‘Diagnosis Unknown’ because they either had a F99 or Z71.1 ICD-10 diagnosis or no diagnosis was recorded. Patients with two or more ICD-10 [26] primary diagnoses over time were included in only one of the above categories according to such hierarchy. For instance, a patient with two primary ICD-10 diagnoses such as F2 and F0 was classified as SSD. Whilst many patients with SSD have other comorbidities such as cannabis misuse [31], given the higher stability of schizophrenia diagnosis over time [32], we chose this category as the first in our hierarchical system. This allowed us to create two clinically meaningful categories to compare: those patients with SSD and those with non-SSD.

**Table 1:** **Diagnoses**

**ICD-10 diagnosis**	**No. completed suicide**	**Diagnostic categories**
F2 ever	54	Schizophrenia spectrum disorder
		54 (17.5%)
Non-F2	197	
F00-09	10	Organic/Drugs
F10-19	35	45 (14.6%)
F31	17	Affective Psychoses
F32.3	6	23 (7.5%)
F32, F33, F34	38	Mood Disorders and Neuroses
F40-48	21	59 (19.2%)
F50-59	1	Others
F60-69	10	33 (10.7%)
Others	22	
F99	16	Diagnosis ‘unknown’
Z71.1	21	94 (30.5%)
ICD-10 diagnosis unrecorded	57	
**Total**	**308**	**308**

ICD-10: International Classification of Mental and Behavioural Disorders [24].

#### Suicide ascertainment and suicide method

Suicide method was ascertained from that recorded in the ONS Certificate of Death [25] (ICD-10 codes) [26] and the following groups were considered: hanging - X83.8, drowning - X71, cutting - X78, poisoning - X64 and Y1, jumping (either from high place or in front of a vehicle) - X80, X81, burning - X76, X77 and undetermined cause of death - Yxx.

#### Health of the Nation Outcome Scale (HoNOS)

The Health of the Nation Outcome Scale (HoNOS) has been a widely used outcome measure in British mental health services since its introduction in the 1990s, found to be a reliable and valid instrument to assess clinical outcomes, and to have easy applicability to clinical settings [22]. HoNOS is formed of 12 items which assess psychiatric symptoms (e.g. depressed mood or hallucinations), alcohol/drugs use and social needs such as relationship problems or daily living activities. Each item is scored on a Likert scale from 0 (absent) to 4 (maximum severity), and the 12 scores are summed to create total scores, i.e. the higher the score, the more complex/severe the case. The last HoNOS recorded for each patient was used for the purposes of this study.

#### Risk assessment

‘Full risk assessment’ is a compulsory target across the Trust when ‘high risk’ is determined from a ‘brief risk assessment’, which is mandatory for all active cases. All patients who have been seen on at least one occasion by a member of the staff have a ‘brief risk assessment’ documented, which is a narrative record of the patient’s risk: i) to one’s self; ii) to others and iii) from others. If the patient is deemed at ‘high’ risk in any of these domains, a ‘full risk assessment’ needs to be completed, which consists of a structured assessment taking the form of present/absent tick-boxes enquiring about widely recognised risk factors for three major clusters: suicide, violence and self-neglect. For this study, only suicide risk assessment was taken into account, which is composed of 15 items (shown in Table 2) including suicidal history, hopelessness, alcohol misuse, living alone or disengagement. Positive responses can be summed to create total scores, i.e. the higher the score the greater the suicide risk [33]. The most recent full risk assessment was considered for these analyses.

**Table 2:** **Risk Assessment: Completion rates, individual items and total scores comparison in SSD and non-SSD suicide completers**

**Completion rate 40 (13.0%)**	**Total sample N = 40**	**SSD N = 19 (19/54 = 35.2%)**	**non-SSD N = 21 (21/254 = 8.3%)**	**ORs (95% CIs) 6.02 (2.94-12.31)**	**Fisher’s exact test P < 0.001**
***Individual items***					p-value
*Suicidal History*	26 (65)	11 (57.9)	15 (71.4)	0.55 (0.15-2.05)	ns
*Lethal Method*	15 (37.5)	5 (26.3)	10 (47.6)	0.39 (0.10-1.49)	ns
*Plan to end life*	8 (20)	2 (10.5)	6 (28.6)	0.29 (0.05-1.68)	ns
***Suicidal ideation***	**16 (40)**	**4 (21)**	**12 (57.1)**	**0.2 (0.05-0.81)**	**0.027**
***Hopelessness***	**18 (45)**	**4 (21)**	**14 (66.6)**	**0.13 (0.03-0.55)**	**0.005**
*Distress*	17 (42.5)	7 (36.8)	10 (47.6)	0.64 (0.18-2.27)	ns
***No control of life***	**15 (37.5)**	**4 (21)**	**11 (52.4)**	**0.24 (0.06-0.98)**	**0.038**
*Alcohol Misuse*	11 (27.5)	7 (36.8)	4 (19.0)	2.47 (0.59-10.40)	ns
***Impulsivity***	**18 (45)**	**5 (26.3)**	**13 (61.9)**	**0.22 (0.06-0.84)**	**0.031**
*Living alone*	18 (45)	8 (42.1)	10 (47.6)	0.80 (0.23-2.80)	ns
*Poor physical health*	9 (22.5)	4 (21)	5 (23.8)	0.85 (0.19-3.79)	ns
***Significant loss***	**18 (45)**	**5 (26.3)**	**13 (61.9)**	**0.22 (0.06-0.84)**	**0.031**
***Disengagement***	**9 (22.5)**	**7 (36.8)**	**2 (9.5)**	**5.54 (0.98-31.25)**	**0.060**
*Recent Discharge from hospital*	11 (27.5)	5 (26.3)	6 (28.6)	0.89 (0.22-3.59)	ns
*Family History*	0	0 (0)	0 (0)	n/a	ns
***Total score***		**4.11 ± 2.88**	**6.24 ± 2.19**		**0.012**

SSD: schizophrenia spectrum disorder; OR: odds ratio; CI: confidence interval; ns: non-significant; in bold: statistically significant differences.

### Statistical analyses

Distributions of continuous variables were inspected by histograms and parametric and non-parametric tests were used in order to examine differences across the above ‘diagnostic categories’ with regard to demographic and clinical variables. Post-hoc analyses investigated inter-groups differences. People dying from suicide with/without SSD were compared in the following respects: i) on demographic and clinical variables using student t tests, Mann–Whitney U tests and Chi-square tests, as appropriate; ii) on suicide methods, calculating odds ratios and adjusting for age at the time of death and ethnicity; iii) on HoNOS, where completed, both individual HoNOS item (Mann–Whitney-U) and total scores (student t-test); iv) on full risk assessment scores for the 40 (13%) patients with this information (t-test). All analyses were conducted using SPSS version 20.0 (SPSS Inc, Chicago, Il, USA).

## Results

Of 120,216 SLaM ‘active’ service users until the 31^st^ December 2011, 308 who died by suicide over 2007–2011 were identified from the SLaM BRC CRIS.

### Sociodemographic and clinical characteristics of the sample

As shown in Table 1, 54 patients (17.5%) had a diagnosis within the schizophrenia spectrum (F2-ICD10). ‘Mood disorders’, including ‘affective psychosis’ (23 patients, 7.5%) and ‘non-psychotic mood disorders and neuroses’ (59, 19.2%), was the most common diagnosis. Also, 45 suicide completers (14.6%) were diagnosed with ‘organic disorders or drugs dependence’ and 33 marginal cases (10.7%) had ‘other’ (known) diagnosis such as Obsessive-Compulsive Disorder (OCD), Attention Deficit Hyperactivity Disorder (ADHD) and Autism Spectrum Disorders (ASD). Of note, 94 patients (30.5%) had no recorded diagnosis, which in most cases (69, 73.4%) was related to having had just one face-to-face contact with the staff (e.g. a single presentation to A&E).

Sociodemographic and clinical characteristics are compared across multiple diagnoses in Table 3, and specifically between those with and without SSD in Table 4. No diagnostic differences were observed in gender; however, age at the time of suicide was younger in those with SSD than the remainder. There were significant group differences in ethnicity across suicide completers’ diagnoses with Black ethnicity more frequent in patients presenting with SSD. Although there were no significant differences in IMD scores across diagnoses (Table 3), these were higher in SSD compared to the remainder. Hearing, visual or mobility impairment did not vary significantly across all diagnoses but mobility impairment was more common in SSD than the remainder. SSD cases had received longer duration of care from the Trust teams and had been seen by a member of the staff substantially more recently before the suicide event.

**Table 3:** **Demographics, clinical and service use-related factors for suicide completers across diagnoses**

	**Total sample 308**	**Schizophrenia spectrum disorder 54 (17.5)**	**Affective psychosis 23 (7.4)**	**Mood disorders/neuroses 62 (20.1)**	**Organic/drugs 45 (14.6)**	**Other diagnoses 30 (9.7)**	**Diagnosis unknown 94 (30.5)**	**p-value**
*Age at the time of death*	**42.7 ± 14.0**	**39.3 ± 11.9**	49.3 ± 14.2	47.0 ± 16.5	44.7 ± 14.0	39.3 ± 13.4	40.4 ± 12.5	0.040
*Gender (males)*	211 (68.5)	38 (70.3)	10 (43.5)	40 (64.5)	36 (80.0)	19 (63.3)	68 (72.3)	0.052
*Ethnicity*								
***White British***	**157 (51)**	**22 (40.7)**	**18 (78.3)**	**45 (72.6)**	**30 (75.0)**	**13 (4.3)**	**29 (30.8)**	**< 0.001**
*Other white background*	30 (9.7)	4 (7.4)	1 (4.3)	3 (4.8)	8 (16.6)	2 (6.6)	12 (12.7)	ns
*South Asian*	5 (1.6)	2 (3.7)	0 (4.3)	2 (3.2)	0 (0)	0 (0)	1 (1.0)	ns
***African and other black***	**25 (8.1)**	**12 (22.2)**	**0 (0)**	**1 (1.6)**	**1 (2.2)**	**1 (3.3)**	**10 (10.6)**	**< 0.001**
***Caribbean***	**11 (3.6)**	**8 (14.8)**	**1 (4.3)**	**0 (0)**	**2 (4.4)**	**0 (0)**	**0 (0)**	**< 0.001**
*Mixed and others*	16 (5.2)	3 (5.5)	1 (4.3)	2 (3.2)	2 (4.4)	4 (13.3)	4 (4.2)	ns
***Missing***	**64 (20.7)**	**3 (5.5)**	**2 (8.7)**	**9 (14.5)**	**2 (4.4)**	**10 (33.3)**	**38 (40.4)**	**< 0.001**
*Deprivation*	28.0 ± 13.7	32.1 ± 11.4	22.5 ± 13.8	27.2 ± 13.5	30.2 ± 13.1	26.1 ± 13.3	27.1 ± 14.3	ns
*Hearing Impairment*	4 (1.3)	1 (1.85)	0 (0)	1 (1.6)	1 (2.2)	0 (0)	1 (1.0)	ns
*Visual Impairment*	2 (0.6)	0 (0)	0 (0)	0 (0)	2 (4.4)	0 (0)	0 (0)	ns
*Mobility Impairment*	8 (2.6)	4 (7.4)	0 (0)	1 (1.6)	0 (0)	1 (3.3)	2 (2.1)	ns
***First language English***	**147 (47.7)**	**34 (62.9)**	**10 (43.5)**	**30 (48.4)**	**28 (62.2)**	**11 (36.6)**	**34 (36.2)**	**0.008**
***Length of service contact (median, days)***	**184**	**1294**	292	184	513	48	**0**	**< 0.001**
*Last face-to-face to death (median, days)*	70	10	19	93	31	177.5	**560**	**< 0.001**

In bold: statistically significant differences.

**Table 4:** **Demographics, clinical and service use-related factors: SSD vs. non-SSD suicide completers**

	**Total sample 308**	**SSD 54 (17.5)**	**non-SSD 254 (82.5)**	**OR (95% CI)**	**p-value**
***Age at death***	**42.7 ± 14.0**	**39.3 ± 11.9**	**43.5 ± 14.4**		**0.048**
*Gender (males)*	211 (68.5)	38 (70.3)	173 (68.1)	1.11 (0.58-2.11)	0.87
***Ethnicity***					
***White***	**187 (60.7)**	**26 (48.4)**	**161 (63.4)**	**0.54 (0.30-0.97)**	**0.046**
***Black***	**36 (11.7)**	**20 (37.0)**	**16 (6.3)**	**8.75 (4.14-18.5)**	**< 0.001**
***Others***	**85 (27.6)**	**8 (14.8)**	**77 (30.3)**	**0.40 (0.18-0.88)**	**0.020**
***Deprivation***	**28.0 ± 13.7**	**32.1 ± 11.4**	**27.1 ± 14.0**		**0.010**
***Mobility Impairment***	**8 (2.6)**	**4 (7.4)**	**4 (0.01)**	**5.00 (1.21-5.66)**	**0.034**
***First language English***	**147 (47.7)**	**34 (62.9)**	**113 (44.5)**	**2.12 (1.15-3.88)**	**0.016**
***Length of service contact (median, days)***	**184**	**1294**	**81**		**< 0.001**
***Last face-to-face to death (median, days)***	**70**	**10**	**146**		**< 0.001**

SSD: schizophrenia spectrum disorder; OR: odds ratio; CI: confidence interval. In bold: statistically significant differences.

### Suicide method

As shown in Tables 5 and 6, ‘hanging’ was the most common suicide method in the sample overall (131, 42.5%). SSD suicide cases had more frequently utilized ‘jumping’, while ‘hanging’ was significantly more frequent among the remainder. There were no suicides by firearms.

**Table 5:** **Suicide method across diagnoses**

	**Total sample 308**	**Schizophrenia spectrum disorder 54 (17.5)**	**Affective psychosis 23 (7.4)**	**Mood disorders 62 (20.1)**	**Organic/drugs 45 (14.6)**	**Other diagnoses 30 (9.7)**	**Diagnosis unknown 94 (30.5)**	**p-value**
***Hanging***	**131 (42.5)**	**14 (25.9)**	**13 (56.5)**	**34 (54.8)**	**17 (37.7)**	**11 (36.6)**	**42 (44.6)**	**0.025**
*Drowning*	21 (6.8)	5 (9.2)	2 (8.7)	5 (8.0)	1 (2.2)	2 (6.6)	6 (6.4)	ns
*Sharp object*	9 (2.9%)	2 (3.7)	0 (0)	1 (1.6)	1 (2.2)	1 (3.3)	4 (4.2)	ns
***Poisoning***	**66 (21.4)**	**7 (12.9)**	**5 (21.7)**	**9 (14.5)**	**17 (37.7)**	**10 (33.3)**	**18 (19.1)**	**0.015**
***Jumping***	**56 (18.2)**	**17 (31.5)**	**3 (13.0)**	**13 (20.9)**	**3 (6.6)**	**5 (16.6)**	**15 (16.0)**	**0.042**
*Burning*	3 (0.9)	2 (3.7)	0 (0)	0 (0)	0 (0)	0 (0)	1 (1.0)	ns
***Undetermined***	**22 (7.4)**	**7 (12.9)**	**0 (0)**	**0 (0)**	**6 (13.3)**	**1 (3.3)**	**8 (8.5)**	**0.025**

SSD: schizophrenia spectrum disorder; OR: odds ratio, CI: confidence interval. In bold: statistically significant differences.

**Table 6:** **Suicide method: schizophrenia spectrum disorder (SSD) vs. non-SSD**

	**Total sample 308**	**SSD 54 (17.5)**	**non-SSD 254 (82.5)**	**ORs (95% CIs)**	**Fisher’s exact test p-value**
***Hanging***	**131 (25.9)**	**14 (25.9)**	**117 (46.0)**	**0.4 (0.2-0.7)**	**0.005**
*Poisoning*	66 (21.4)	7 (12.9)	59 (23.2)	0.5 (0.2-1.1)	ns
***Jumping***	**56 (18.2)**	**17 (31.5)**	**39 (15.3)**	**2.5 (1.3-4.9)**	**0.008**
*Cutting*	9 (2.9)	2 (3.7)	7 (2.7)	1.4 (0.3-6.7)	ns
*Burning*	3 (0.9)	2 (3.7)	1 (0.4)	9.7 (0.9-109.4)	0.081
*Drowning*	21 (6.8)	5 (9.2)	16 (6.3)	1.5 (0.5-4.4)	ns
*Undetermined*	22 (7.4)	7 (12.9)	15 (5.9)	2.4 (0.9-6.1)	0.091

SSD: schizophrenia spectrum disorder; OR: odds ratio; CI: confidence interval; ns: non-significant. In bold: statistically significant differences.

### Health of the Nation Outcome Scale (HoNOS)

116 suicide completers (37.6%) had at least one HoNOS completed. Completion rates differed across diagnoses: 83.3% of those with SSD; 35.0% of the remainder (OR = 12.9, 95% CI 6.0-27.7, p < 0.001). On individual items, patients with SSD scored significantly higher on ‘hallucinations & delusions’, while subjects with non-SSD had higher scores on ‘depressed mood’ and ‘self-injury’ (Table 7).

**Table 7:** **Health of the Nation Outcome Scale (HoNOS): Completion rates, individual items and total scores comparison in SSD and non-SSD suicide completers**

**Completion rate 116 (37.6%)**	**SSD 45 (45/54 = 83.3%)**	**non-SSD 69 (69/254 = 35.0%)**	**p < 0.001 OR = 12.89 (6.00-27.73)**
***Individual items***			p-value
*Agitated behaviour*	0.44	0.51	ns
***Self-injury***	**0.18**	**1.03**	**<0.001**
*Drinking & Drugs*	0.71	0.70	ns
*Cognitive Problems*	0.31	0.59	ns
*Physical Illness*	0.82	0.68	ns
***Hallucinations/Delusions***	**1.22**	**0.29**	**<0.001**
***Depressed Mood***	**0.89**	**1.80**	**<0.001**
*Other mental problems*	1.38	1.65	ns
*Relationship problems*	1.07	1.17	ns
*Daily living*	0.87	0.87	ns
*Living*	0.67	0.59	ns
*Occupational problems*	0.96	0.78	ns
***Total score***	9.55	10.52	ns

SSD: schizophrenia spectrum disorder; OR: odds ratio; CI: confidence interval; ns: non-significant. In bold: statistically significant differences.

### Full risk assessment

As described in Table 2, only 40 suicide completers (13%) had at least one full risk assessment completed: 19 SSD (35.2%) vs. 21 non-SSD (8.3%) (OR = 6.0, 95% CI 2.9-12.3, p < 0.001). Mean ± SD total scores were lower in SSD (4.1 ± 2.9) compared to non-SSD (6.2 ± 2.2; t = −2.65, p = 0.012). With regard to individual items, patients who had SSD receiving a full risk assessment were less likely to be recorded as having ‘suicidal ideation’, ‘hopelessness’, ‘lack of control over life’, ‘impulsivity’ or a ‘significant loss’; while ‘disengagement’ was more common in individuals with SSD at borderline statistical significance.

## Discussion

### Principal findings

In a large clinical case register sourced from electronic mental health records linked to a national death certification database, we investigated characteristics of patients dying from suicide, comparing these between those with and without SSD. Four major conclusions can be drawn from our results.

First, a population of secondary mental healthcare users who ended their lives over the study period (2007–2011) was identified from our case register. Nearly one in five of the suicide completers had been diagnosed with schizophrenia spectrum disorders (SSD), which is approximately double that in previous reports [8], although this may reflect the high prevalence of psychotic disorders in South-East London [34] and our methodology does not permit us to calculate a suicide rate in each diagnostic category.

Second, several differences were found in sociodemographic characteristics between SSD and other diagnoses. Specifically, those subjects with SSD died at a younger age, they tended to be more commonly of Black origin and they were more socially deprived than non-SSD suicide completers, which replicated previous findings in our catchment area with non-suicide samples [35,36]. Given the study methodology, particularly the lack of a control non-suicide group, no causality conclusions can be inferred from the above findings. Thus, while some previous studies have shown an association between psychosis and Black ethnicity [34], other groups have raised concerns regarding a diagnostic bias towards psychosis in Black people [37]. However, previous studies from our group, which carefully considered the above potential bias in the methodology, revealed a link between Black Ethnicity, social deprivation and psychosis in our catchment area [31]. Hence, our findings concerning differences between patients with and without schizophrenia spectrum disorders who died by suicide in terms of social deprivation and ethnicity might reflect specific sociodemographic characteristics of our population irrespective of such a fatal outcome despite previous literature showing a relationship between ethnicity and suicide [38]. Nevertheless, further research is warranted in this area.

Third, with regard to suicide method, we found that while hanging was the most common suicide method in the whole sample, patients with SSD were more likely to have used ‘jumping’ (from an height or in front of a vehicle) to take their lives, consistent with previous literature on ‘suicide completion and method’ [4,11,39,40]. This may have implications for suicide prevention. Limiting availability of lethal methods has been demonstrated to reduce suicide rates at a population level [4]. Also, restricting access to suicide hotspots such as heights through safety barriers (e.g. [10]) and railway lines by installing platform edge doors [41] has been reported to reduce overall suicide rates at such places [42]. In keeping with the above, no suicides by gunshot were identified in our sample, which is likely to be due to the restrictions to firearms access in the UK. Hence, by linking our findings concerning suicide method and diagnosis to previous literature there are grounds to speculate that installation of physical barriers in bridges, tall buildings and railway stations near psychiatric hospitals may prevent patients with SSD from suicide. Nonetheless, further research should be conducted in this area.

Fourth, it seems that clinicians deem patients who have SSD at greater risk of suicide and they are therefore more likely to have received a full risk assessment, as well as having a higher likelihood of HoNOS completion, which is relatively in contrast to previous literature [20]. However, the above instruments may have been completed due to concerns raised regarding other risks such as violence and/or self-neglect.

Interestingly, known suicide risk factors tended to be less commonly recorded as present in those who have SSD than in subjects with non-SSD. Of relevance, disengagement was found to be (significantly) more frequent in SSD than non-SSD suicide completers, however. Despite the above, half of suicide completers with a SSD had been seen by a member of the staff shortly before such a fatal outcome, which is in line with previous studies showing the relative inability of clinicians to predict imminent suicide risk in individuals with SSD under their care [43].

With regard to HoNOS items in our sample of suicide completers, while symptoms severity such as depressed mood and ‘hallucinations & delusions’ differed across diagnostic groups, as expected, interestingly ‘social needs’, ‘cognitive deficits’ or ‘alcohol/drugs problems’ did not (significantly) vary between patients with SSD and without SSD. In this regard, it could be speculated that the care package provided to these patients by the Trust succeeded in meeting their complex social needs and accordingly the most recent HoNOS scores regarding social needs did not reveal differences across the above diagnostic categories. An alternative explanation, which could not be tested in this study due to using a sample of suicide completers without control group, might be that there are no associations between (met/unmet) social needs, psychiatric diagnosis and suicidality. In addition, we replicated the role of ‘depression’ and history of self-injury in suicide completion, both irrespective of diagnosis and across the different diagnoses [44], including SSD [18,45].

Regarding classic risk factors, we replicated the role of suicidal history and suicidal ideation, hopelessness, impulsivity and significant loses in suicide risk irrespective of diagnosis [44,46] being highly prevalent, although they were more frequent in the non-SSD group.

Hence, it seems that overall suicide might be less predictable, and therefore less preventable, in patients with SSD than in those without [18,47,48]. However, disengagement was specifically more common in the SSD group than in all-other diagnoses. Hence, there are grounds to consider that insight, which has been associated with adherence [49], might be a protective factor for suicide in schizophrenia spectrum disorders [18] despite common assertions to the contrary (see [50] for review). In addition, stigma, which prevents patients from receiving proper care [51], may play a relevant role in suicide risk in patients under secondary mental health services who disengage, particularly among those with SSD [52]. Nevertheless, more research is needed to test whether anti-stigma campaigns can reduce suicide rates both at a population level and in secondary mental health services users.

### Strengths and limitations

This study focused on the rare outcome of suicide completion and therefore adds to previous findings from samples of suicide attempters (e.g. [53]). Moreover, by using a large case register linked to national mortality data, all those patients receiving secondary mental healthcare in our catchment area who died by suicide over 2007–2011 were included in the study with the only exception of those who ended their lives outside the UK. Since only a small proportion of patients living in South-East London receive private mental health care, our sample is likely to be representative. In addition, a wide range of demographic and clinical variables, including service use-related factors and specific scales such as HoNOS and ‘risk assessment’, were analyzed.

However, several limitations should be borne in mind when interpreting our results. First, the sample was formed of secondary mental health services users living in south-east London, an inner-urban area, and results may not generalise to people receiving mental health input from primary care or those in rural areas. Second, HoNOS and risk assessment ratings were available for only a small percentage of the whole sample, and there were clear differences between diagnoses in completion rates. Also, we can speculate that those patients without SSD who had HONOS and risk assessment completed were deemed ‘at-high-risk’ patients by their clinical teams. In addition, although just the last HONOS and risk assessment were considered for the analyses, the time from that assessment to dying from suicide should be taken into account when drawing associations of causality since risk factors evaluated by those instruments may have changed over the study period. Hence, our findings regarding HONOS and risk assessment ratings differences across diagnoses should be considered cautiously. In keeping with the above, it should be noted that a wide range of variables have been taken over a prolonged period of time, which also varies across the study patients, who ranged from having one single assessment to several years under secondary mental healthcare, thus reflecting the real-world nature of our data. However, the two main variables of interest for this study, namely a SSD diagnosis, which has been demonstrated to have a high diagnostic stability over time [32], and suicide completion can be considered independent of time.

## Conclusions

Our findings show that patients with SSD who end their lives have usually been deemed by their clinicians as being at greater need of risk assessment and accordingly they tend to be more closely monitored, including completion of full risk assessment and HoNOS. However, such evaluated ‘known’ risk factors for suicide appear to be more relevant in those patients with non-SSD. Moreover, most of the individuals who had SSD included in this study had been seen by a mental health professional shortly before dying by suicide, jumping being the most common suicide method.

A successful approach for suicide prevention is likely to require a combination of both population-level strategies such as restricting access to lethal means and measures focused on high-risk groups such as patients with SSD. Specifically, our results suggest that the classic suicide risk assessment and prevention approach in secondary mental healthcare may have little relevance for patients with SSD.

## Abbreviations

WHO: World Health Organization
HONOS: Health of the nation outcome scale
SSD: Schizophrenia spectrum disorder
SLaM: South London & Maudsley NHS foundation trust
BRC: Biomedical research centre
CRIS: Clinical record interactive search
ONS: Office for National Statistics
ICD-10: International classification of mental and behavioural disorders
IMD: Index of multiple deprivations
